# Transylvania University

**Published:** 1834

**Authors:** 


### VIII. Transylvania University.

This institution still remains, in point of numbers, the second medical school in the Union. The class of the present session (including nine resident graduates) amounts to 246.

